# Inflammatory Mediators in Periodontal Pathogenesis

**DOI:** 10.1155/2019/2610184

**Published:** 2019-06-18

**Authors:** Olivier Huck, Nurcan Buduneli, Denisse Bravo

**Affiliations:** ^1^INSERM (French National Institute of Health and Medical Research), UMR 1260, Regenerative Nanomedicine, Fédération de Médecine Translationnelle de Strasbourg (FMTS), Strasbourg, France; ^2^Université de Strasbourg, Faculté de Chirurgie-Dentaire, 8 Sue Sainte-Elisabeth, 67000 Strasbourg, France; ^3^Department of Periodontology, School of Dentistry, Ege University, İzmir, Turkey; ^4^Laboratorio de Microbiología Oral, Facultad de Odontología, Universidad de Chile, Santiago, Chile

This special issue is dedicated to the periodontal pathogenesis and emphasizes the complexity of the disease. This topic has received interesting original studies or review articles, and a total of 12 manuscripts were finally accepted out of the 21 manuscripts received. The selected articles address different aspects of periodontal disease, from the implication of selected cytokines or gene polymorphism on periodontitis. Among the others, the role of receptors such as TLR-4 and CXCR2 and inflammatory and immune-related markers such as CD14, OPG, RANKL, or IL-18 was demonstrated. Identification of specific inflammatory patterns may help to better understand the different phases of periodontitis onset and progression and may also lead to the development of new clinical approaches for the diagnosis and monitoring of periodontitis patients.

Systemic influence of periodontitis is also discussed in this special issue. Periodontal diseases are regarded as a significant risk factor for various systemic diseases such as cardiovascular diseases, diabetes, or adverse pregnancy outcomes. Here, some of the suggested links are discussed with innovative approaches. Indeed, F. Ceccarelli et al. detailed the potential molecular mechanisms underlying the association between periodontitis and rheumatoid arthritis from genetic factors and autoantibodies to inflammatory biomarkers, while S. Schulz et al. emphasize the association of genetic variations in proinflammatory cytokines (TNF-*α*, IFN-*γ*) and cytokine receptor (IL4R*α*) in rheumatoid arthritis and periodontal diseases. Another article by A. Hoare et al. presented the role of chronic inflammation driven by periodontitis-associated bacteria on the development of oral and extraoral carcinogenesis. These data clearly emphasize the importance of periodontal diagnosis and appropriate treatment not only for a healthy dentition but also for a systemic health.

Different treatment procedures are also investigated in an attempt to correlate periodontal treatment outcomes and systemic levels of inflammatory biomarkers. The effect of Er,Cr:YSGG and diode lasers on IL-37 and IL-1*β* levels was analyzed by A. C. Talmaç et al. in the context of aggressive periodontitis treatment. The role of CEMP-1 during the early phase of healing was analyzed by C. Dellavia et al., and C. Petit et al. reviewed the pleiotropic effects of statins and their potenital interest in the management of periodontal diseases.

Periodontal diseases are among the most common chronic inflammatory and infectious diseases worldwide, and we hope that this special issue brings new insights into the complex mechanisms driving the inflammatory processes associated with such bacteria-elicited disease. These valuable data may help to develop new diagnostic tools and therapeutic strategies based on the control of the inflammatory and immune responses acting in the pathogenesis of periodontal diseases. Moreover, it can be suggested that a closer collaboration between dental professionals and physicians may help to improve the health as a whole.

